# On the Structure, Functions, and Diseases of the Liver; and on the Action of Chalogogue Medicines

**Published:** 1852-09

**Authors:** C. Handfield Jones


### SELECTIONS.

From the London Lancet.

*On the Structure, Function, and Diseases of the Liver; and on the Action of Cholagogue Medicines.* By C. Handfield Jones, M.D, F.R.S.

The author first described the minute structure of the liver, which consisted essentially of a mass of nucleated cells or celloid particles, usually more perfectly formed than the cells either of the salivary or renal glands, presenting a distinct nucleus, with a nucleolar spot, an exterior envelope, and an included mass of soft, semi-solid, albuminous substance, which commonly contained a few oily molecules. In addition to these, in well-nourished livers, were numerous free nuclei, imbedded in albuminous blastema, which exhibited various stages of progress towards the mature or perfect cell. The oily contents of the cells were subject to great variation, both in the same individual and in different classes of animals;—the less perfect the type of the respiratory process, the greater the quantity of oily matter in the hepatic cells. The cells in their general mass constituted the hepatic parenchyma; this might be subdivided into smaller portions, called lobules, which were separated from each other more or less completely by fissures, the fissures themselves being continuous with canals that ramified throughout the parenchyma, and which, from containing the portal vein and its associated vessels, had been termed portal canals. In reference to the mode of distribution of the vessels, originally so well expounded by M. Kiernan, the author remarked that he decidedly agreed with Theile, who denied the existence of the vaginal branches and plexuses of the portal vein mentioned by M. Kiernan. The author quoted from a paper by Mr. Paget, who had described these vaginal plexus to be derived, not from the portal veins, but from the hepatic arteries, from which they were completely filled, when both arteries and veins were at the same time injected. The interlobular portal veins were therefore derived directly from the portal veins; and those which appeared to be vaginal branches of the portal vein were its internal roots, by which it received the blood which had served for the nutrition of the hepatic ducts and other vessels of the liver. After alluding to the mode of ramification of the hepatic artery, and the divisions of the hepatic ducts following the branches of the portal canal, the author referred to the relation which existed between the ultimate ducts and the cells constituting the parenchyma of the lobules. The prevalent opinion had been, that these cells were exactly homologous to the cells of the renal tubuli or salivary vesicles, like them growing on a free surface open to the exterior.

Hence some anatomists had believed that they had detected a basement membrane forming anastomosing tubes, constituting a true lobular biliary plexus. Others, unable to find a basement membrane, had described the ducts as continued into the parenchyma of the lobules, as channels without proper walls, mere intercellular passages. After referring to the researches and opinions of Weber, Muller, Professor Retzius, on the one side, and of Val Guillon, Gerlach, and Dr. Carpenter, on the other, the author stated that the views of Kolliker, who denied the existence of intercellular passages into the lobules, agreed very nearly with his, (the author's,) and conceded his main position, that the cavity of the ducts was quite shut off from the cells of the lobules or their interspaces. The structure of the ultimate ducts, which the author had first discovered, was peculiar, and seemed to indicate strongly that they exerted active functions, and that they were something more than mere afferent canals. The injection of the duct, in the livers of pigs, by the double method, using separately saturated watery solutions of bichromate of potass and acetate of lead, exhibited an abundant yellow precipitate in the fissures; but in very few parts did it penetrate the lobules, which must have happened if there existed a lobular biliary plexus of intercellular passages. The author conceived, therefore, that the hepatic ducts did something more than merely carry out already elaborated bile. The ultimate ducts were far too small, and too sparingly distributed, to be able to take up the bile from so vast a mass of cells as that which constituted the parenchyma. If the ducts did not extend beyond the margins of the lobules, of which the author had no doubt, then the bile must be transmitted from cell to cell; or there was a march of cells outwards from centre to the circumference; or else the bile, arriving at the margin of the lobules, was taken up by the ultimate ducts in some unknown way. The author thought such assumptions groundless and unnecessary; and that the pathological state of fatty liver, as well as the fatty liver occurring naturally in fishes, showed that the secretion of the parenchyma was not identical with that of the ducts, for the gall-bladder could hardly contain deep green bile, when the parenchyma was nought but a mass of oil. He concluded, then, that the parenchymal cells of the lobules did not merely secrete bile which was carried off unaltered by the ducts, but that the cells secreted biliary material, or some of its components, which were not fully elaborated or formed into perfect bile, except by the action of the ultimate ducts. Proof was then offered that the hepatic cells did not ordinarily contain bile, although it was commonly held they did. He believed that to be a diseased or exceptional condition, not found in the hepatic cells of slaughtered or healthy animals. Furthermore, a yellow tint in the cells was no proof of the presence of bile; it showed merely the presence of pigment, and yellow pigment is found in the fat of some animals, quite independent of biliary secretion. Chemistry must be resorted to, to solve the question of the presence of bile in the hepatic cells. The author had made alcoholic extracts of the livers of different animals, and having evaporated to dryness, the residue, when dissolved in water, failed to show by Pettenkoffer's test, any reaction characteristic of the presence of the bile. The author, however, did not wish to express a positive opinion, but he thought that the received opinion had need of more direct evidence, before it could be regarded as proved. He then detailed the mode in which the morphological structure of the ultimate biliary duct fulfilled the function of the secretion. The chemical changes which the ultimate ducts effected, might be conceived according to the hypothesis of Lehmann; and a summary of our present knowledge might stand as follows: sugar, oil, and a yellow pigment were found in the parenchyma of the liver; bile is not found there, but in the ducts; it is inferred, then, that the ducts, through their ultimate extreme portions, *make* the bile. The author next proceeded to detail some experiments made relative to the action of cholagogue medicines, the results of which led him to believe that mercury, muriate of manganese, and colchicum, were the only ones which seemed to increase the production of yellow pigmentary matter in the cells of the liver. They also increased the production of glyco-cholite and tauro-cholite of soda; but it had to be determined whether the quantity of these principles was always proportionate to the yellow pigment. It was clear that the cholagogue action of a medicine, its emulging effects on the ducts, was distinct from that which it excited in the production of biliary pigment. One very important effect of the administration of mercury on the liver, was noticed to be congestion of this organ; an argument rather forbidding the use of the remedy in inflammation of the substance of the liver, a plan otherwise recommended by analogical experience. The author then proceeded to the subject of diseases of the liver; the microscopic appearances of fatty liver were detailed, and the question, what constituted true fatty degeneration of the liver, discussed. Was it a simple increase in the quantity of oil naturally existing in the hepatic cells, or was it a further and more important change? He believed the latter. In the liver of animals artificially fed on oily food, and subsequently examined, the cells, as well as the intercellular substance, were loaded with oil-molecules; the accumulation of oil was equal everywhere. But in the morbid state of fatty degeneration, the oil drops were not enclosed in distinct cells, but appeared to lie in an indistinct and granular, or semi-fibrous substratum. Another point of difference consisted in the absence of sugar in true fatty degeneration; while in the liver of an animal fed on oily food to produce a fatty liver, sugar could be detected. Another point of importance was the limitation of fatty degeneration to the margin of the lobules; it was not a mere accumulation of oil in the marginal cells, but a destruction of those cells: a liver thus affected presented the lobules marked out by a zone of opaque matter. No satisfactory explanation of this tendency of oil to accumulate in the marginal cells could be offered. Fatty degeneration of the liver might occur in very different diseases; it was by no means peculiar to phthisis Reference was then made to the waxy liver of Rokitansky, with which the author was not sure that he was acquainted. Cirrhosis was then mentioned, and Rokitansky's description quoted, as also that of Dr. Budd, whose views expressed the opinion ordinarily received, but from which the author in some degree dissented. The author believed that an unhealthy nutritive process was the essence of cirrhosis, and might be developed in one of three situations. 1. In the larger and moderate-sized portal canals, excluding only the smallest. 2. In these last and in the fissures. 3. In the smaller canals and fissures, and in the substance of the lobules. The first form produced common *hobnail* liver; the second and third, the tough, firm, dense liver, sometimes termed brawny. The author considered cirrhosis to represent essentially a degenerative process, and to arise from the effusion of an unhealthy plasma, not only in the canals and fissures, where it induced unnatural increase, but also in the external part of the lobules. where it passed into a solid form, and constituted an unmorpho-granular substance, compressing the capillaries and obstructing the secreting cells. The thickening and condensation of the fibrous tissue in the liver were thus not so much the effect of an inflammatory action, as of a low degenerative process, analagous to that which stiffened the valves of the heart and contracted the orifices; and which view the author thought was supported by the results exhibited in a table appended to the paper. The subject of jaundice next received attention. This was a disease that manifestly resulted from the conveyance into the blood of bile pigment, a constituent of the bile which was essentially excrementitious, and intended to be cast out with the fæcal matter. In many cases it existed only as retained excretion; in others it seemed to be formed in excessive quantity, as in the acute yellow atrophy of the liver. Yellow matter was often found in the central cells of the lobules, and, nevertheless, there was no jaundice. It should be borne in mind that the yellow pigment, as it existed in the cells, did not evidence the presence of biliary matter, of cholic acid, or its conjugates. The yellow matter could be extracted by alcohol, and its characteristic reaction obtained by nitric acid, but Pettenkoffer's test decided against the presence of any organic biliary acid. The deep color of the urine in jaundice depended on the presence of bile pigment solely; no trace of cholic acid was discoverable. The author considered the majority of cases of jaundice to depend on the absorption into the blood, not of completely formed bile, but of one of its constituents only, the yellow pigment: and this might take place in one of three ways: 1, by a mechanical obstruction to the flow of bile into the intestine, through the ductus communis choledochus; 2, from inaction of the elaborating ducts; 3, with or without impairment of the action of the excretory ducts, when an increased quantity of yellow pigment was formed in the parenchyma of the liver.

